# Development, implementation and feedback for an online speciality membership examination in orthodontics during the COVID-19 pandemic

**DOI:** 10.1038/s41415-021-3535-5

**Published:** 2021-10-22

**Authors:** Charlotte E. Eckhardt, Jadbinder Seehra, Stephen M. Chadwick, Kim Voerman, Alex Landau, Fiona S. Ryan, Padhraig S. Fleming, Matthew Garrett, Martyn T. Cobourne

**Affiliations:** 1 Lead Cleft Lip and Palate Orthodontist, Consultant Orthodontist, Maxillofacial Department, Morriston Hospital, Swansea Bay University Health Board, Swansea, UK; 2 Senior Clinical Teacher and Consultant Orthodontist, Centre for Craniofacial Development & Regeneration, Faculty of Dentistry, Oral & Craniofacial Sciences, King´s College London, London, UK; 3 Consultant Orthodontist, Orthodontic Department, Countess of Chester Hospital, Chester, UK; 4 Quality Assurance Manager, Royal College of Surgeons of England, London, UK; 5 Director of Dental and Surgical Examinations, Royal College of Surgeons of England, London, UK; 6 Consultant Orthodontist, Department of Orthodontics, Royal National ENT and Eastman Dental Hospitals, University College Hospitals London NHS Trust, London, UK; 7 Professor of Orthodontics, Barts & The London School of Medicine and Dentistry, Queen Mary University of London, London, UK; 8 Consultant in Restorative Dentistry, Royal National ENT and Eastman Dental Hospitals, University College Hospitals London NHS Trust, London, UK; Dean, Faculty of Dental Surgery, Royal College of Surgeons of England, London, UK; 9 Professor of Orthodontics, Centre for Craniofacial Development & Regeneration, Faculty of Dentistry, Oral & Craniofacial Sciences, King´s College London, London, UK; Chair of Examinations, Faculty of Dental Surgery, Royal College of Surgeons of England, London, UK

## Abstract

**Introduction** The Royal College of Surgeons of England (RCSEng) and the Royal College of Physicians and Surgeons of Glasgow (RCPSG) offer the bi-collegiate Membership in Orthodontics (MOrth) examination, a summative assessment of specialist knowledge, skill and behaviour in orthodontics. The COVID-19 pandemic has had a profound global effect on almost every facet of normal life, including the conduct of face-to-face examinations. We highlight development, implementation and feedback for the bi-collegiate MOrth Part 2 examination delivered remotely to a cohort of candidates in September 2020 by RCSEng/RCPSG.

**Methods** Two anonymised online surveys (Google Forms) were distributed via electronic mail following completion of the examination diet. Forty-two candidates were sent a survey covering four domains and comprising a total of 31 questions. The 20 examiners were sent a survey containing eight questions. In both surveys, free-text responses were also collected. A rating system was used to categorise responses. All survey responses were summarised in an online data collection sheet.

**Results** The response rate was 78.5% (33/42) and 75% (15/20) for candidates and examiners, respectively. Overall, favourable responses in relation to all sections of the assessment were elicited from candidates with the majority (mean 79.8%; 75.8-81.9%) reporting that the online examination format worked well. Equally, favourable responses were reported by examiners. Notably, 80% of examiners felt that the online exam style did not affect the mark a candidate would receive, and 100% were confident that the marks the candidates received were a reflection of their ability and were not affected by the online delivery of the assessment.

**Conclusions** The feedback from both candidates and examiners relating to an online remote assessment of the bi-collegiate MOrth Part 2 was generally positive. Based on the survey responses, this format of a high-stakes examination was acceptable to all stakeholders, and demonstrated a high level of perceived validity and reliability in terms of content.

## Key points

- Highlights development, implementation and feedback for a remote bi-collegiate specialty examination in orthodontics.
- Candidates responded favourably in relation to all sections of the assessment, with the majority reporting that the online examination format worked well.
- Examiners were confident that the marks candidates received reflected their ability and were not affected by online delivery of the assessment.
- Remote delivery of a high-stakes specialty examination is a valid and reliable method of assessment, facilitating trainee progression in an environment of social distancing.

## Introduction

In the United Kingdom (UK), specialist training in orthodontics is conducted through approved three-year full-time speciality training programmes following the Speciality Advisory Committee and General Dental Council-approved orthodontic curriculum.^1^ Knowledge and competency is evaluated using both formative and summative assessment, which contributes to the evidence portfolio for entry onto the specialist list in orthodontics. Formative assessments are mapped to the curriculum and conducted throughout training, contributing to an annual review of competency progression.^2^ Trainees are also required to undertake original research as part of the process of training. The Membership in Orthodontics (MOrth) examination of the surgical Royal Colleges is a summative assessment of specialist knowledge, skill and behaviour. Currently, three dental faculties run diets of the MOrth examination - the Royal College of Surgeons of England (RCSEng)^3^ and the Royal College of Physicians and Surgeons of Glasgow (RCPSG)^4^ combine to offer the bi-collegiate Membership in Orthodontics (Bi-MOrth) examination,^3^ while the Royal College of Surgeons of Edinburgh (RCSEd) offers the MOrth RCSEd. The MOrth examination is traditionally undertaken on a face-to-face basis in designated examination centres with both candidates and examiners present.

The ongoing coronavirus (COVID-19) pandemic has multiple potential implications for face-to-face examinations, including requirements for medical shielding, social distancing, infection control, testing, safety issues for candidates and examiners, and potential redeployment issues for all participants. These issues can, in turn, impact on ongoing postgraduate specialist training, with a potential lack of formative assessment opportunities impairing competency-based training programmes.^5^ Following national lockdown in March 2020, all Royal College assessments were postponed as part of the general restrictions aimed at slowing viral spread. Against this backdrop, the Statutory Education Bodies of the four nations recommended that where possible, progression through clinical training should continue without detriment to trainees.^6^ Discussions between the UK Royal Colleges and higher education stakeholders (UK Committee of Postgraduate Dental Deans and Directors [COPDEND],^7^ Health Education England [HEE])^8^ resulted in an agreed position that while Royal Colleges should plan to deliver examinations in a face-to-face COVID-19-secure environment from September 2020 onwards, they should also make contingency plans for remote assessment.

To facilitate continued assessment during the pandemic, two options were available: 1) face-to-face examinations utilising multiple locations to follow social distancing regulations; or 2) remote examinations using video conference calling platforms.^6^^,^^9^ The first option can be hindered by resource-related factors, including the identification of multiple COVID-19-safe venues across the UK and associated increased costs. Moreover, it would be contrary to government recommendations to expect groups of candidates and examiners to travel to various regions when recommendations were in place to limit group numbers, contact and travel. In addition, there may be significant consequences for any candidate or examiner who develops symptoms of COVID-19 while attending a face-to-face examination at a distant location. The use of advanced online technology has been advocated as an aid to delivering training outcomes^10^ because in-person assessments can be curtailed by social distancing measures.^6^ Remote online assessments in medical education are not a new concept and it has been recognised for some time that they have potential advantages over traditional forms of assessment. These include instant feedback for the learner, the ability to monitor learner progress in real time, and a move away from the artificial distinctions between formative and summative assessment towards a programme of assessment for learning.^11^ These generic advantages are complemented by the lack of required travel for both candidates and examiners, the lack of restrictions on candidate numbers and no requirement for examination venues.^6^^,^^11^

This article highlights development, implementation and subsequent feedback for the RCSEng and RCPSG Bi-MOrth Part 2 high-stakes speciality summative assessment delivered remotely for the first time on 14-17 September 2020.

## Reframing the RCSEng/RCPSG Bi-MOrth examination for remote delivery

Prior to the pandemic, the Bi-MOrth examination took place over a number of days in a single examination centre, with candidates sitting a written paper and examiners meeting them face-to-face during the presented and unseen cases and objective structured clinical examination (OSCE) sections. Candidates would travel from across the UK and internationally to attend the examination. By the beginning of July 2020, a final decision was made by RCSEng/RCPSG to run the Bi-MOrth assessment online and remotely. This decision was driven by the fact that potential candidates needed certainty as to how the examination would be run and confidence that their training and career progression would not be hindered. At the time, there was also a high level of uncertainty regarding travel restrictions across the UK and from international locations. If a face-to-face examination had been planned, this would potentially be at odds with UK or overseas government advice.

It is imperative to any reframing of an assessment format that the domains of validity, reliability and fairness are maintained.^6^^,^^12^ Establishing the psychometric properties of an assessment is complex. Validity refers to whether the test in question measures what it purports to measure, which includes content and also the cognitive processes under assessment. Reliability refers to the degree to which scores from a particular test are reproducible from one use of the test to the next, as well as the internal consistency of results across items within a test. Fairness refers to the concept of impartiality and just treatment of candidates to ensure that marks achieved are a reflection of their responses and are not related to irrelevant characteristics. Reframing the Bi-MOrth format involved the repurposing of some currently used examination formats (Table 1). Four principal formats were deemed appropriate (Appendix 1): 1) short-answer questions (SAQs); 2) presented cases; 3) communication stations; and 4) structured clinical reasoning (SCR; unseen cases). The reframing was undertaken by a core group of senior examiners with experience in question-setting in conjunction with college educationalists. The aim was to ensure that there was no substantial compromise in the core assessment aims of the examination, while moving to an online format. In order to maintain content validity, blueprinting to the orthodontic speciality training curriculum was consistently applied.

**Table 1:** Traditional and reframed formats of the Bi-MOrth examination. Prior to the pandemic, the MOrth examination took place over a number of days in a single examination centre, with candidates sitting the written paper and examiners meeting them face to face during the presented and unseen cases and OSCE sections. Candidates would travel from across the UK and internationally to attend the examination

RCSEng and RCPSG intercollegiate membership examination in orthodontics (Bi-MOrth)			
Traditional Bi-MOrth format (face-to-face assessment)	Reframed Bi-MOrth format (remote assessment)	Design and adjustment	Domains assessed
Combined MCQ and SAQ written paper	SAQ written paper only	Existing SAQ and knowledge-based OSCE questions from the question bank were modified or used as a template for question design	Knowledge Application of knowledge
OSCE circuit Knowledge stations Communication (four stations with independent actors) Practical stations	Knowledge stations (re-purposed into SAQ written paper) Communication station (replaced by two communication scenarios) Practical stations not included	Examiners acted the roles of actors in each communication scenario All other components of communication stations were maintained as per face-to-face assessment	Assimilation of information Application of knowledge Communication
Presented cases	Maintained	Candidates submitted their cases electronically in advance Cases were accepted if they were nearing completion +/- incomplete records If uncompleted cases were presented, then viva to include questions and discussion concerning potential finishing procedures, approaches to analysis of outcomes using cephalometric superimposition and other techniques, and discussion of approaches to retention	Treatment planning Practical assimilation of information Application of knowledge Communication
Structured clinical reasoning/unseen cases Candidates provided with clinical photographs, representation of study models, radiographs and cephalometric tracings	Maintained	Physical models were not available for review and direct measurements on related models could not be undertaken Case templates included photographs of models and documentation of key inter-arch measurements All other components of structured clinical reasoning/unseen cases were maintained as per face-to-face assessment	Assimilation of information Application of knowledge Communication Treatment planning
Key: MCQ = multiple-choice questions; SAQ = short-answer questions;			

Another substantial difference between the current and revised Bi-MOrth Part 2 format was removal of the OSCE section. This would normally comprise of 16 stations with a minimum of 13 assessing five domains. Remote online delivery of an OSCE, specifically with the retention of its existing practical elements, was not thought to be feasible and hence the elements usually covered in the OSCE were repurposed into the revised examination sections (Table 2).

**Table 2:** Attributes assessed in the OSCE component of Part 2 Bi-MOrth

Attributes assessed in OSCE	Re-purposed in reframed Part 2 Bi-MOrth
Good communication skills	Communication stations, unseen and presented cases
Ability to analyse and interpret diagnostic information and material	Assessed in the unseen, presented cases and SAQs
Demonstrate practical skills normally undertaken as part of clinical practice	Assessed in WBA (not formally assessed in overseas candidates)
Interpret and appraise data from publications	SAQ
Apply appropriate decision-making in clinical situations	Unseen cases, presented cases and SAQs
Key: SAQ = short-answer questions; WBA = workplace-based assessments.	

The Bi-MOrth examination in its current format allows compensation between all sections of the assessment. This is based on the premise that the relationship between blueprinting of the curriculum and the competencies tested within different elements of the assessment is not distinct. The utility of the assessment is improved by having the more valid and reliable written/verbal paper and the more valid but less reliable presented cases both contributing to the overall mark. The pass mark is less reliable if candidates are able to pass sections of the assessment independently, because this means the whole assessment is only as reliable as the least reliable element. Similarly, in the reframed COVID-19 format, there was compensation between all three components.

For this assessment, it was important to re-create a remote face-to-face environment to allow delivery of interactive sections of the examination. Telemedicine and, in particular, video conference calling have increased in popularity within healthcare.^13^ The perceived limitations of telemedicine include technical issues, costs, additional training and lack of interaction.^14^ There are multiple applications available, but in relation to examination requirements (presented cases, SCR and communication scenarios), Microsoft Teams (MS Teams; Microsoft Inc. 2017) was selected as the preferred platform due to its functionality and accessibility.^15^^,^^16^ This system allows the transfer of information securely because it can be used in collaboration with NHS mail and multiple video screens can be visible at the same time, permitting visual interaction with both candidate and examiner. There are no limits to meeting duration, which allows flexibility in the planning of examination section durations. Specific teams can be created within an organisation to simulate virtual examination rooms, backgrounds can be blurred to reduce distractions, documents such as SCR records can be uploaded and accessed, and multiple individuals can participate in the same meeting. The latter lends itself to candidate and examiner briefing or debriefing sessions. Examiners or candidates can also be invited into virtual rooms or meetings at specific times, which enables a degree of control in the timetabling of assessments. Candidates were invigilated by remote proctoring for the written element and in real time by staff and examiners for the oral element.

## Candidate and examiner feedback

Forty-two candidates sat the September diet of the Bi-MOrth Part 2 examination between 14-17 September 2020 (compared to 21 candidates for the May 2019 diet), with a total of 20 examiners. Invitation e-mails containing links to two online surveys devised for this examination (Google Forms) were distributed six days following the diet. Candidates were asked to complete a survey covering four domains (individual aspects of the examination, overall satisfaction, areas of good practice, concerns) comprising 31 questions. This questionnaire formed part of the normal process of obtaining objective feedback from candidates undertaking college examinations. All information was handled anonymously in accordance with General Data Protection Regulation. Examiners were also asked to complete a survey containing eight questions. This included domains similar to the candidate survey, but also explored examiner perception of the reframed MOrth compared to the traditional format. In both surveys, the option of providing anonymous free-text responses was provided. Surveys were developed from existing RCSEng examination feedback forms and modified following consensus agreement between a panel of examination team members.

A rating system was used to highlight favourable (strongly agree/agree), neutral and unfavourable (disagree/strongly disagree) responses, respectively. The free-text responses were analysed and discussed qualitatively using a general thematic content analysis by three authors. Demographic variables were not collected in either survey in order to preserve anonymity. All survey responses were summarised in an online data collection sheet. Invitations to complete the surveys were only sent once.

## Results

The online surveys were completed and returned by 33 candidates and 15 examiners, reflecting a response rate of 78.5% (33/42) and 75% (15/20), respectively. Only descriptive statistics were used to summarise survey responses.

### Candidate survey

Overall, favourable responses (strongly agree/agree) predominated in relation to all sections of the assessment (Tables 3, 4, 5 and 6). The SAQ written paper was felt to be fair (66.7%) and consisted of questions relevant to the candidates' taught courses (81.8%), although only around half agreed that the questions asked were expected from what had been taught (51.5%). Approximately one-third of candidates agreed that there was enough time to complete the SAQ paper (36.3%). For the interactive components of the assessment - case presentations, communication stations and unseen cases, respectively - the summative favourable responses for the following questions were as follows: 'The examiners seemed friendly': 66.7% (unseen cases) and 81.8% (case presentations); 'Were the questions from the examiners clear?': 67.4% (unseen cases) and 84.8% (case presentations); 'Were you asked questions you felt were relevant to the presented material?' (mean 89.7%; range 84.8-97.0%); 'Enough time was given for the discussion' (mean 87.8%; range 78.7-93.9%); 'Did you feel comfortable during the discussion?' (mean 70.7%; range 63.6-75.8%); 'The sound quality was to a good standard' (mean 83.8%; range 81.8-87.8%); 'The video quality was to a good standard*'* (mean 80.9%; range 78.8-82.0%); and 'The online examination format worked well' (mean 79.8%; range 75.8-81.9%).

**Table 3:** Percentage of candidate responses for the SAQ section (n = 33)

SAQ	Strongly agree	Agree	Neutral	Disagree	Strongly disagree
The exam seemed fair	21.2%^*^	45.5%^*^	27.3%^**^	3.0%^†^	3.0%^†^
Were the questions asked expected from what you had been taught?	30.3%^*^	51.5%^*^	18.2%^**^	-	-
Enough time was given to complete the exam	12.1%^*^	24.2^*^	30.3%^**^	15.2%^†^	18.2%^†^
The online exam software was easy to use	15.2%^*^	33.3%^*^	27.3%^**^	12.1%^†^	12.1%^†^
Did you feel dissatisfied with the question format?	9.1%^†^	12.1%^†^	27.3%^**^	39.4%^*^	12.1%^*^
Did you feel the staff had properly briefed you on the exam format?	36.4%^*^	27.3%^*^	24.2%^**^	9.1%^†^	3.0%^†^
Did you feel adequately supported by the staff during the exam?	51.5%^*^	24.2%^*^	15.2%^**^	6.1%^†^	3.0%^†^
Key: * = favourable responses. ** = neutral responses. † = unfavourable responses.

**Table 4:** Percentage of candidate responses for the case presentations section (n = 33)

Case presentations	Strongly agree	Agree	Neutral	Disagree	Strongly disagree
The examiners seemed friendly	39.4%^*^	42.4%^*^	9.1%^**^	9.1%^†^	-
Were the questions from the examiners clear?	33.3%^*^	51.5%^*^	6.1%^**^	3.0%^†^	6.1%^†^
Were you asked questions that you felt were relevant to your presented material?	36.4%^*^	60.6%^*^	-	3%^†^	-
Enough time was given for the discussion	24.2%^*^	69.7%^*^	3.0%^**^	-	3.0%^†^
Did you feel comfortable during the discussion?	30.3%^*^	42.4%^*^	18.2%^**^	9.1%^†^	-
The sound quality was to a good standard	33.3%^*^	54.5%^*^	12.1%^**^	-	-
The video quality was to a good standard	39.4%^*^	39.4%^*^	15.2%^**^	3.0%^†^	3.0%^†^
The online examination format worked well	36.4%^*^	45.5%^*^	9.1%^**^	6.1%^†^	3.0%^†^
Key: * = favourable responses. ** = neutral responses. † = unfavourable responses.

**Table 5:** Percentage of candidate responses for the communication section (n = 33)

Communication	Strongly agree	Agree	Neutral	Disagree	Strongly disagree
Did you understand the actor?	42.4%^*^	45.5%^*^	6.1%^**^	6.1%^†^	-
Were the questions from the actors clear?	33.3%^*^	54.5%^*^	3.0%^**^	9.1%^†^	-
Were you asked questions you felt were relevant to the presented material?	24.2%^*^	60.6%^*^	15.2%^**^	-	-
Enough time was given for the discussion	39.4%^*^	51.5%^*^	3.0%^**^	6.1%^†^	-
Did you feel comfortable during the discussion?	27.3%^*^	48.5%^*^	15.2%^**^	3.0%^†^	6.1%^†^
The sound quality was to a good standard	33.3%^*^	48.5%^*^	15.2%^**^	3.0%^†^	-
The video quality was to a good standard	36.4%^*^	45.5%^*^	9.1%^**^	6.1%^†^	3.0%^†^
The online examination format worked well	30.3%^*^	45.5%^*^	15.2%^**^	3.0%^†^	6.1%^†^
Key: * = favourable responses. ** = neutral responses. † = unfavourable responses.

**Table 6:** Percentage of candidate responses for the unseen cases section (n = 33)

Unseen cases	Strongly agree	Agree	Neutral	Disagree	Strongly disagree
The examiners seemed friendly	27.3%^*^	39.4%^*^	24.2%^**^	6.1%^†^	3.0%^†^
Were the questions from the examiners clear?	33.3%^*^	48.5%^*^	9.1%^**^	9.1%^†^	-
Were you asked questions you felt were relevant to the presented material?	33.3%^*^	54.5%^*^	12.1%^**^	-	-
Enough time was given for the discussion	24.2%^*^	54.5%^*^	12.1%^**^	9.1%^†^	-
Did you feel comfortable during the discussion?	21.2%^*^	42.4%^*^	24.2%^**^	12.1%^†^	-
The sound quality was to a good standard	30.3%^*^	54.5%^*^	12.1%^**^	3.0%^†^	-
The video quality was to a good standard	24.2%^*^	57.6%^*^	9.1%^**^	3.0%^†^	6.1%^†^
The online examination format worked well	33.3%^*^	48.5%^*^	15.2%^**^	3.0%^†^	-
Key: * = favourable responses. ** = neutral responses. † = unfavourable responses.					

The free-text responses obtained from candidates are grouped into four main themes (Table 7). A few candidates appeared to have issues relating to connectivity, which impacted upon sound and video reliability. Functionality issues related to the Surpass software,^17^ which was used to deliver the written SAQ section, were also expressed by candidates. However, candidates were very positive about the support they received from the examination staff and the examination overall.

**Table 7:** Thematic grouping of candidates' free responses (SAQ = short-answer questions)

Theme	Responses
Technical issues with internet	'My connection was disrupted, and I had to restart my laptop and phone twice' (SAQ) 'As long as a good internet connection it was good quality' (case presentation) 'Minor sound issues - had some feedback noise as I was talking' 'Sometimes I missed words that had been said due to internet quality, but I just asked for the question to be repeated or confirmed and therefore was not a major issue' 'Lost connection and had to relocate mid-discussion' 'I did have a short drop out internet connectivity on my end for one minute - it didn't really cause any major problems though' 'One of the examiner's video and sound froze during the exam, which was off-putting, but the other examiner dealt with the situation well' 'Video quality - I had an issue on the last day of the examinations and I was not able to see the examiners. I found this made the exam harder and it would have been nice to see the examiners. I appreciate that it isn't a fault of the examination and is a problem with the online format'
Software issues	'Help from the proctor exam online messenger service was very prompt and reassuring when my page hadn't loaded' (SAQ) 'Unable to click directly to the question flagged' (SAQ) 'Unable to scroll through numbers, mine kept reverting back to screen share option so wasted time and increased stress' (SAQ) 'When examiners blurred their background, it was a little off-putting to only see half their head at times' 'I had a bit of a technical issue during one of my communication scenarios, which meant that the conversation was quite stilted' 'Needing to maximise and minimise the screen took up time during the preparation' (unseen cases) 'Some difficulties navigating the screen to see the case and examiners, but managed to overcome it in subsequent cases' (unseen cases)
Support from staff	'The support up to the exam was good as this was all explained to us and having the testing sessions was great help to put us at ease' 'RCS staff were all very friendly and helpful' 'Examiners were very friendly and encouraging in general' 'RCS support were great and practice sessions prior were very helpful'
Overall	'Well-run exam, very friendly and supportive administrative team, kept us informed of everything' 'Worked well' 'Felt strange but workable alternative given our circumstances' 'Overall, I am very happy that I got the opportunity to sit MOrth in September and appreciate how much work must have gone into setting this all up. Thank you'

### Examiner survey

Mirroring the candidate survey, favourable responses were evident for all questions (Table 8). The summative favourable responses for the following questions were as follows: 'Enough time was given for the discussion element of the assessment' (93.3%); 'The sound quality was to a good standard' (93.3%); 'The video quality was to a good standard' (93.3%); 'The online examination format worked well' (93.4%); 'Did you feel that the online exam style affected the mark a candidate would receive?' (80% disagree); 'Did you feel as if proper precautions were taken with the exam in response to the coronavirus pandemic?' (100%); and 'Do you think a face-to-face examination would have been more advantageous for the candidate?' (60.0% disagree). Importantly, for the final question - 'I am confident that the marks the candidates received were a reflection of their ability and were not affected by the online delivery of the assessment' - 100% of examiners gave a favourable response.

**Table 8:** Percentage of examiners' responses (n = 15)

Question/statement	Strongly agree	Agree	Neutral	Disagree	Strongly disagree
Enough time was given for the discussion element of the assessment	80.0%^*^	13.3%^*^	-	6.7%^†^	-
The sound quality was to a good standard	53.3%^*^	40.0%^*^	-	6.7%^†^	-
The video quality was to a good standard	40.0%^*^	53.3%^*^	-	6.7%^†^	-
The online examination format worked well	66.7%^*^	26.7%^*^	6.7%^**^	-	-
Did you feel that the online exam style affected the mark a candidate would receive?	-	6.7%^†^	13.3%^**^	20.0%^*^	60.0%^*^
Did you feel as if proper precautions were taken with the exam in response to the coronavirus pandemic?	93.3%^*^	6.7%^*^	-	-	-
Do you think a face-to-face examination would have been more advantageous for the candidate?	6.7%^†^	13.3%^†^	20.0%^**^	26.7%^*^	33.3%^*^
I am confident that the marks the candidates received were a reflection of their ability and were not affected by the online delivery of the assessment	66.7%^*^	33.3%^*^	-	-	-
Key: * = favourable responses. ** = neutral responses. † = unfavourable responses.					

The free-text responses from examiners have been grouped into three main themes (Table 9). Again, issues related to internet connectivity were stated but examiners were also positive about the support they received from the examination staff as well as their perception of the examination overall. Individual responses regarding the time taken to score and apparent differences between online remote and face-to-face examinations were also expressed.

**Table 9:** Thematic grouping of examiners' free responses

Theme	Responses
Technical issues with internet	'The most stressful part was getting online' 'A few candidates had connection difficulties' 'Sound and video quality were variable and not always consistent'
Support from staff	'Occasional IT dropouts but this was very well managed by the examinations team' 'All worked very well with excellent back-up from the examination team' 'Very well organised. Smooth movement between rooms as the detailed timetable was very helpful' 'It ran better than I thought due to excellent team at RCS'
Overall	'The exam was excellent under the circumstances. In-person assessment does, however, remain the gold standard with better interaction, responsiveness and lower risk of problems in normal times' 'The online exam was extremely well set up and managed. However, it was definitely not the same as a face-to-face exam. A lot of non-verbal cues were lost and this sometimes disrupted the flow of the vivas' 'Worked extremely well and allowed all aspects that would usually be examined to be so' 'Score collation was more difficult and time consuming' 'The fact we used formats already established by other examinations gave us a lot of confidence we could make it work for our assessment' 'A thoroughly well-run and equivalent exam process. Allowed for the full range usually examined to be assessed in a similar manner. A fair, well-run and robust examination that safe-guarded the standards of the IMOrth and put the candidates at the heart of the process. In a time of such uncertainty, it was a great achievement to be able to supply an appropriate examination to allow candidates to progress in their careers' 'I think we have been part of revolution in assessment; however, the future may involve the integration of the face-to-face assessment with the online assessment rather than the replacement of one form of assessment with the other' 'Fantastic job by all. The right thing to have done in the circumstances but I feel the exam lost the special sense of occasion due to the online format. I don't think this is something that could ever be recreated without a face-to-face format - ideally in the college' 'Ran really well'

## Discussion

This article outlines the processes undertaken by RCSEng/RCSPG to develop and implement an online remote high-stakes Bi-MOrth examination during the COVID-19 pandemic, and subsequent candidate and examiner feedback relating to the process.

Currently, limited evidence from high-stakes online remote assessments involving postgraduate students exists. However, a recent study reporting the effectiveness of an online examination taken by final-year dental students has been published.^18^ Consistent with our evaluation, the exam was run without any untoward events, with both students and examiners expressing satisfaction with the process overall.^18^ Interestingly, some similar issues to those raised in our evaluation were reported. Some students felt they did not have enough time during the written paper, and they had problems with software functionality and some technical issues. For examiners, issues included a negative increase in time to grade the assessment and technical issues. In the current evaluation, a disadvantage of online remote assessment compared to face-to-face cited by one examiner was summarised: 'It was definitely not the same as a face-to-face exam. A lot of non-verbal cues were lost, and this sometimes disrupted flow of the vivas'. This opinion has been previously reported, with the absence of face-to-face interactions denying the opportunity to observe or demonstrate soft skills.^9^

It is well established that undertaking high-stakes assessment is associated with increased stress and anxiety for students.^19^ Indeed, this may lead to differences in students' own self-assessment of performance compared to their actual performance,^20^ which can influence evaluation responses. For example, the perception that one-third of candidates felt there was not enough time to complete the SAQ paper appears to be at odds with the fact that additional time had been allocated for this section. However, it appears from the evaluation responses that the reframed online remote Bi-MOrth was met with a high degree of acceptability from both candidates and examiners. Although not assessed in this evaluation, it is known that previous experience of online learning is associated with increased satisfaction with online assessment.^18^ It should also be considered that the online survey responses were collated before the candidates were informed of their exam results. This increases the validity of this evaluation as it allowed candidates to reflect on their experience on a different day and under the assurance that all completed and returned surveys were to remain anonymous.

The criteria (reliability, validity, impact on future learning and practice, acceptability to stakeholders and costs incurred) for assessing the utility of an assessment have been previously described.^21^ It is reassuring that the vast majority of candidates (89.7%) felt they were asked questions relevant to the presented material, that examiners disagreed that the online exam style affected the mark a candidate would receive (80%) and that examiners were confident that the marks the candidates received were a reflection of their ability and were not affected by the online delivery of the assessment (100%). Although not formally assessed, these findings support the assumption that both content validity and perceived reliability were maintained in this assessment. Both candidates and examiners expressed a high level of satisfaction in the post-evaluation feedback, suggesting high levels of acceptability to stakeholders. Although not measured, it is anticipated that the costs for the diet would be considerably less compared to face-to-face examinations, as the majority of examiners and all of the candidates did not have to travel to an examination centre. Costs associated with examiner subsistence would be reduced accordingly and candidates did not have to book additional accommodation or travel.

We have highlighted the merits of online remote assessment in challenging circumstances. However, the examiner free-text responses did suggest that some reservations remain: 'The exam was excellent under the circumstances. In-person assessment does, however, remain the gold standard with better interaction, responsiveness and lower risk of problems in normal times' and 'Fantastic job by all. The right thing to have done in the circumstances but I feel the exam lost the special sense of occasion due to the online format. I don't think this is something that could ever be recreated without a face-to-face format - ideally in the college'. It must be stressed that if a safe face-to-face assessment could have been delivered, this would have been the first choice. However, due to the ever-changing COVID-19 landscape, this would not have been feasible without accepting a high level of risk for candidates, examiners and the examinations team. In the long term, it is unclear if online remote assessment will become a direct replacement for face-to-face examinations or complementary to traditional methods. Clearly, further prospective studies comparing the assessment methods against defined outcomes are required. In the immediate future, until the COVID-19 risk has dissipated, online remote assessments will be continued and refined in order to deliver high-stakes examinations across both dental and medical specialities. Technical problems, largely associated with internet connectivity and software, remain an issue but are manageable with appropriate contingency planning including safeguarding measures such as use of back-up phone hotspots, additional time allocated to an assessment if connectivity is lost and appropriate training of stakeholders. The importance of adequate support and training from examination staff in facilitating online remote assessments was particularly apparent in the surveys of both stakeholder groups.

Concerns regarding both security and IT reliability have been expressed in relation to online assessments.^6^ To ensure the assessment was conducted in a secure manner, the following measures were implemented by college examination support staff before the candidate completed the online sections: 1) pre-checks to ensure the candidates system was properly set up and secure; 2) candidate authentication via name and candidate number verification; and 3) the local environment was checked by asking the candidate to share real-time views of their immediate location via their computer. In addition, all examination material accessible during the examination was password-protected. To reproduce the process of quality assurance, two senior examiners were given access to all virtual examination rooms. Prior to the assessment, both candidates and examiners were briefed that an additional examiner may be observing their oral examination. In addition, the Surpass^17^ software used for the SAQ records the candidate through two cameras and monitors their screen usage throughout the examination. Recordings of the written section were also reviewed by examination staff following the diet and before the release of results to identify any potential suspicious candidate behaviour.

In the event of IT failure or disruption during the assessment, a protocol was in place to ensure no candidate would be disadvantaged. Examiners were instructed to inform the examination support staff immediately if issues arose. Methods to facilitate communication included the use of the messaging function in MS Teams, messages sent via a WhatsApp group communication that was end-to-end encrypted (created specifically for examiners and administrated by examination staff) and direct telephone contact. If time was lost during the examination due to connectivity issues, candidates were briefed that this time would be added onto their session to ensure parity was maintained. Additionally, within the examination timetable, extra time was allocated between candidates to account for delays. Candidates and examiners were also provided with extensive training sessions either individually or as a group in the weeks before the diet. These sessions consisted of tutorials and demonstrations describing interaction with and navigation through virtual rooms created within MS Teams, with delineation of the examination process and explanation on alerting the examination team concerning issues arising. The software also allowed examiners to discuss the submitted presented cases as well as the unseen case material in advance of the examination diet.

There are, of course, wider implications of remote online examinations in an increasingly technology-dependent world. The COVID-19 pandemic has shown us how education and assessment processes need to be able to continue in an environment of social distancing. We believe that this online diet of a dental speciality examination in orthodontics has demonstrated that it is possible to undertake high-stakes examinations while maintaining the safety of all participants, and that these approaches are likely to be applicable to all areas of dentistry and wider medicine.

## Conclusions

The main goal of this reframed Bi-MOrth assessment was to provide a robust equitable exit examination in orthodontics and facilitate career progression for orthodontic specialist trainees in a time of unprecedented crisis. Conforming to government guidelines and protecting both candidates and examiners from the risk of infection transmission were key considerations. Reframing of the assessment, instigation of quality and assurance processes, selection of appropriate video conference calling platforms, and provision of training and support to both candidates and examiners facilitated the delivery of a valid, robust and reliable high-stakes speciality summative assessment. Feedback from candidates and examiners was generally positive. The timing of resumption of traditional high-stakes assessments in a normal face-to-face manner remains uncertain. The present study indicated that in accordance with both COPDEND and HEE guidance, the RCSEng successfully delivered a high-stakes examination in orthodontics that was acceptable to all participants.

**Appendix 1 Format of the re-structured MOrth examination** **SAQs**

The written component of the original MOrth Part 2 consisted of a three-hour combined multiple-choice question and short-answer question (SAQ) written paper. Students who have passed the written component of a recognised university training programme are exempt from a written element in normal circumstances. This section aims to assess the candidate's applied knowledge and understanding of orthodontic theory. In the new format, the written paper was revised to a two-hour open-ended SAQ paper consisting of 12 questions. To ensure validity, reliability and fairness, existing SAQ and knowledge-based OSCE questions from the question bank were modified or used as a template for question design. Numbers of questions and timing was based on other successful examinations that use this format and blueprinting to the current orthodontic curriculum. The exemptions currently in place for the written element of Part 2 MOrth were removed in the re-structured assessment, with all candidates sitting the SAQ section. However, candidates who had successfully completed the MOrth RCSEd written paper were offered an exemption.

**Presented cases**

Presented cases assess clinical management skills of the candidate through the written presentation of three treated clinical cases, which have contributed significantly to their professional development during their training. These attributes are tested both by assessment of the written case material provided by the candidate and by their ability to answer related questions in a viva voce. Candidates submitted their cases electronically in advance and these were then assessed by examiners prior to the exam diet. Two cases selected by the examiners were discussed in a 30-minute online oral examination with the candidate. Clinical case records are accompanied by a signed statement from the accredited clinical supervisor and Training Programme Director confirming the involvement of the candidate in the treatment of the selected cases. In view of the disruption to clinical services in the UK during lockdown, cases were accepted if they were nearing completion even if the records were incomplete, as the emphasis of the oral examination was to assess if the candidate could demonstrate an understanding of the case and to evaluate the outcome of the care they had provided (Table 2). The lack of completed cases was offset by discussion concerning potential finishing procedures, approaches to analysis of outcomes using cephalometric superimposition and other techniques, and discussion of approaches to retention.

**Communication stations**

In the revised format, two rather than the normal four communication stations that are normally assessed in the OSCE section of the examination were included. These are designed to explore the candidate's ability to communicate information (usually to a patient or parent), their knowledge and the application of that knowledge. The candidates are provided with a scenario and given time to assimilate the information. An actor is also given the same scenario with specific questions to ask the candidate and an examiner is present to mark the candidate against a set list of criteria. While communication is observed and assessed in all verbal interactions with the candidate, the construction of the communication stations differs from that of both the unseen and presented cases. The revised online format necessitated the use of examiners as actors, with marks allocated for content and delivery of information allied to related communication skills. However, all other elements common to the traditional examination were maintained.

**SCR (unseen cases)**

This section is designed to assess the candidate's breadth and depth of knowledge concerning diagnosis, treatment planning and critical thinking skills. It consists of discussions around the advantages and disadvantages of alternative treatment options and examination of knowledge of orthodontic theory and technical expertise. To ensure validity, reliability and fairness, for all structured clinical reasoning (SCR) cases, the following was undertaken: 1) all SCRs were standard set in terms of quality of records and possible different treatment options by all examiners prior to the diet; 2) candidates were presented with case records (four cases) in a standardised format (involving clinical photographs, representation of study models, radiographs and cephalometric tracings) demonstrating a variety of orthodontic problems; and 3) the terminology and language used in the SCR case template has been consistently used in previous diets, both in the UK and internationally.

SCR cases include routine orthodontic cases of moderate complexity or more severe cases involving multidisciplinary management. Candidates are allocated 15 minutes to examine the records followed by a 15-minute oral examination, focusing on diagnosis and treatment planning, treatment mechanics and the management of the malocclusion for each case. In order to reduce duplication during the oral examination, examiners are instructed to concentrate on different aspects of each case, with the aim of increasing the amount of the syllabus covered in the assessment. Virtual delivery dictated that physical models were not available for review and direct measurements on related models could not be undertaken. As such, models were presented electronically with consistent delineation of key inter-arch measurements, including midline positions and overjet.
